# An Effective Cache Algorithm for Heterogeneous Storage Systems

**DOI:** 10.1155/2013/693845

**Published:** 2013-12-16

**Authors:** Yong Li, Dan Feng, Zhan Shi

**Affiliations:** School of Computer Science and Technology, Wuhan National Lab for Optoelectronics, Huazhong University of Science and Technology, Wuhan 430074, China

## Abstract

Modern storage environment is commonly composed of heterogeneous storage devices. However, traditional cache algorithms exhibit performance degradation in heterogeneous storage systems because they were not designed to work with the diverse performance characteristics. In this paper, we present a new cache algorithm called HCM for heterogeneous storage systems. The HCM algorithm partitions the cache among the disks and adopts an effective scheme to balance the work across the disks. Furthermore, it applies benefit-cost analysis to choose the best allocation of cache block to improve the performance. Conducting simulations with a variety of traces and a wide range of cache size, our experiments show that HCM significantly outperforms the existing state-of-the-art storage-aware cache algorithms.

## 1. Introduction

**Figure 1:**
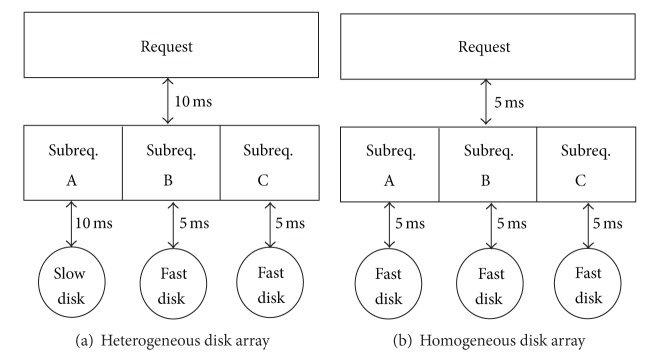
The typical I/O request in heterogeneous and homogeneous disk array, respectively.

Data center always uses a flexible approach to build highly scalable storage systems for rapidly growing data, such as storage virtualization technology. Such large scalable storage systems are commonly composed of heterogeneous storage devices, with a large number of storage devices of different types like Integrated Drive Electronics (IDE) and Serial AT Attachment (SATA) and new storage devices added over time for extending capacity and replacing failed storage devices. Device with relatively low performance may become the bottleneck in such a heterogeneous environment, given that the data placements of these systems are always configured to stripe pattern for parallelism. However, the parallelism has a new problem in such heterogeneous storage environment. If an I/O request accesses multiple disks with different performance, the response time of the request is close to the response time on slow disk. That is because the access to fast devices is faster than access to slow devices. So, the fast devices have to wait for the completion of the subrequest accessed on the slow devices, which will introduce waiting time for fast devices when a request accesses multiple devices with diverse performance. We illustrate it by an example shown in Figure 1. In this example, the disk array is composed of three disks and an accessed request is split into three subrequests. We assume that the response time of the fast device is 5 ms and the slow device is 10 ms. In the homogeneous disk array, the finial response time is 5 ms. However, in the heterogeneous disk array, the final response time is 10 ms due to waiting for the completion of the access on the slow devices.

Many cache policies have been widely used to alleviate the performance gap between I/O systems and processor. Heterogeneous storage systems, however, present a new challenge to those cache policies. Traditional cache algorithms are cost-oblivious, treating all blocks as if they were accessed with the same cost. This assumption is far from being valid for devices of diverse performance characteristics, in which the requests are more likely to congest in the slowest devices. Obviously, this will cause an imbalance in a heterogeneous storage system and will result in the degradation of the overall performance.

### 1.1. Background and Motivation

Recently, several cost-aware algorithms have been proposed to address the above problem, such as Forney's algorithm [1] and Liton's algorithm [2]. Both of their cache algorithms are based on aggregate partitioning which assigns one partition to each device. The allocation among the divided partitions is adaptive with the delay of each partition.

The basic idea of these cost-aware cache algorithms for heterogeneous storage systems is to decrease access delay of the bottleneck partition by allocating more cache blocks. The efficiency of adaptive allocation among divided partitions is affected by two factors.

1. *Utility of cache block*. The relationship between cache size and hit ratio is not linear after a certain threshold of cache size [2]. It means that cache hit ratio will not increase significantly once cache size is beyond the threshold. In contrast, removing a cache block from partition below the threshold may significantly decrease hit ratio. So, how to identify the utility of a cache block for different partition is very important.
2. *Balance cache blocks across partitions*. It is necessary to maintain balance cache blocks across partitions, because a partition with small cache size may become the new slowest partition, which resulted in a decrease in overall performance. For example, assume there are two disks with different performance, and the slow disk is working under sequential workload and the fast disk is working under random workload. Because of the workload difference, the slow partition has smaller delay and gets less cache blocks. However, if the workload changes from sequential to random, the cache size of the slow partition will be far below the demanded size. This will result in sharp increase in access delay.

However, Forney and Liton's algorithms focus on only one of two factors discussed above. Together these two factors inspire our algorithm to further improve the performance.

### 1.2. Our Contribution

In this paper, we propose a novel cost-aware cache algorithm for heterogeneous storage system, which focuses on both factors discussed above. We apply cost-benefit analysis to allocate cache blocks where they will have the greatest utility. Here, cost refers to the increase in delay of a partition when shrinking a cache block. Benefit refers to the decrease in delay of a partition when adding an extra cache block. The change in utility of allocation can be described as the difference between benefit and cost value. We estimate the impact of each alternative allocation on the divided partitions and then choose the one with the greatest utility which has maximum benefit and minimum cost. Furthermore, we propose a simple but effective approach to address the imbalance problem. The algorithm in this approach sets a lower bound of cache size for each partition and dynamically adjusts the lower bound according to the change of workload. The shrinking of cache block is bounded by the lower bound. It helps prevent heavy performance degradation as the change of workload and maintain stable performance for all partitions.  Table 1 summarizes the three groups of cache algorithms discussed above.

## 2. The HCM Cache Algorithm

HCM is a partition-based algorithm and divide the cache into several partitions for different devices which are managed separately. The key problem in the partition-based algorithm is how to effectively allocate the cache blocks among the partitions. In HCM algorithm, there are two schemes to the allocation of the cache blocks: continuous reallocation and periodic reallocation scheme. The continuous reallocation scheme evicts the cache block with minimum utility for the demand access. Many researches [2–4] had shown that the relationship between the cache size and hit ratio is nonlinear if beyond certain threshold. So, the performance benefit of addition cache blocks depends on the workload characteristics of the class. For example, the cache block allocated to the frequently accessed block will obtain more benefit than that allocated to the little accessed block. Thus, it can be beneficial in terms of performance to allocate cache blocks by their utility. To determine the utility of cache blocks, we construct a benefit-cost model based on access pattern. The periodic reallocation scheme focuses on how to balance cache blocks across partitions. As discussed above, the partition size also plays an important role in the overall performance. The allocated cache blocks will not yield immediate performance benefits. The performance benefits come true only if they have cache hits in future. However, the partition size may sharply decrease as the change of workload characteristic, like temporal burst of accesses on other partitions. This will lead to the cache blocks with more utility which are earlier evicted before being accessed. So, it is important that maintaining the provision of appropriate cache blocks for each partition achieves stable performance.

**Figure 2:**
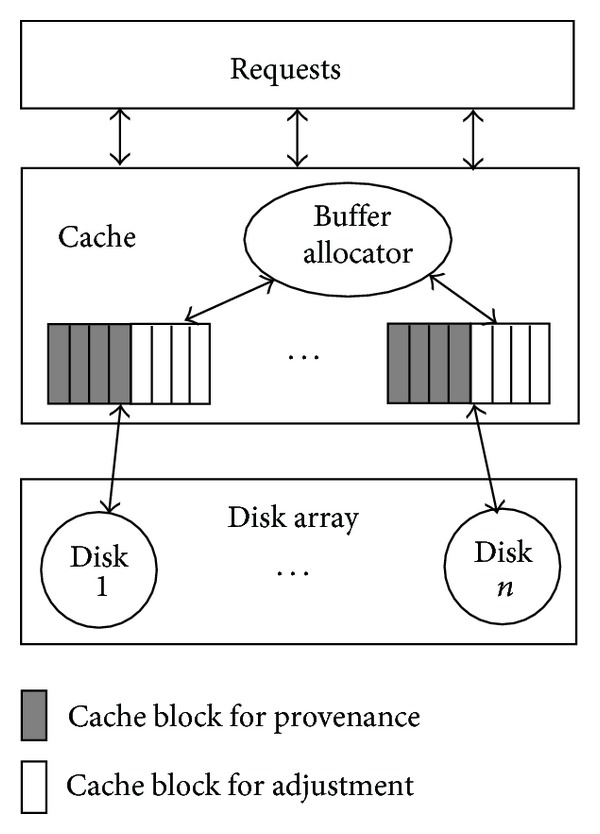
The high-level architecture of our algorithm.

Figure 2 illustrates our high-level architecture. The cache is explicitly divided into *n* partitions among disks *D*_1_,…, *D*_*n*_ and then *i*th disk's data is only stored in the *i*th partition. Partitions are managed separately and possible to employ any previously cache replacement policies including Least Recently Used (LRU), Least-Frequently Used (LFU) and LRU-K [5]. Different policies for different partitions can also be used. The cache space in the partition is split into two areas. One is the provision for stable performance, which we called provision area. The size of provision area is computed periodically by periodic reallocation scheme. The other part is called adjustment area. The cache block in the adjustment area is allocated by the continuous reallocation scheme. The cache block allocation of the adjustment area is more active than the provision area. The continuous reallocation scheme will allocate a cache block to a partition once we find opportunities to improve the utility of the cache block. That is because the more early the decision is taken, the more accuracy the prediction will be. Thus, the size of adjustment area is adapted dynamically to the change of workload. It is necessary to carefully determine the size of both areas. Because a smaller size in provision area may lead to unstable performance and a smaller size in adjustment area may lead to low utility of cache blocks. Both of them may make the degradation of overall performance.

### 2.1. Continuous Reallocation

Continuous reallocation scheme is based on cost-benefit analysis. When cache is full, the allocation of partition-based scheme should first decide which partition is the best candidate for eviction and then use general cache replacement policy to select the evicted block, such as LRU, Clock. Our algorithm classifies partitions into two categories according to the delay of partition: consumer that have larger delay and suppliers that have smaller delay. Consumer takes a cache block from supplier when the cache is full. Our algorithm estimates the benefit of giving a cache block to a consumer and the cost of taking a cache block from a supplier. Here, the benefit refers to the estimation of performance increase in future with addition of an extra cache block and cost refers to the estimation of performance decrease in future with shrinking of a cache block. The goal of our algorithm is selecting the allocation with the greatest utility, which refers to the biggest difference between the benefit and cost.

#### 2.1.1. Estimate Cost

The access delay of an I/O request can be described as expression (1), where *H*(*n*) is the expected hitratio using *n* cache blocks, *T*_cache_ is the time for fetching from the cache, and *T*_disk_ is the time for fetching from disk:

(1) $$\begin{array}{c} T\left(n\right)=H\left(n\right)T_{\text{cache}}+\left(1-H\left(n\right)\right)T_{\text{disk}}. \end{array}$$

The shrinking of a partition will decrease the hit ratio and introduce extra cost because of the increment in number of I/O operations. The cost can be computed as the difference in access delay in future accesses, as expression (2) shows. For simplicity, we ignore *T*_cache_ as it is much smaller than *T*_disk_:

(2) $$\begin{array}{c} \Delta T=T\left(n-1\right)-T\left(n\right)=\Delta H\left(n\right)T_{\text{disk}}, \end{array}$$

where Δ*H*(*n*) is the increase in hit ratio if the cache increases by a single block. Our algorithm classifies requests into three access patterns: random, sequential, looping, and computing Δ*H*(*n*) individually. Similar estimation can be found in [6–8]. For a random reference with the length of *R*, it can be considered as a uniform distribution with *R* blocks. If the cache size *n* > *R*, the access will be all hit in the cache. Thus, the hit ratio is 1 and the Δ*H*(*n*) is 0. If the cache size *n* ≤ *R*, the hit ratio is *n*/*R*. Thus, the Δ*H*(*n*) is Δ*H*(*n*) = *n*/*R* − (*n* − 1)/*R* = 1/*R*. For sequential references, any given block will never be referenced again. So, the hit ratio is 0 and so is Δ*H*(*n*). For a looping reference with reference length *L* and loop length *l*, the hit ratio is min⁡(*l*, *n*)/*L*. If *l* ≥ *n*, the Δ*H*(*n*) is Δ*H*(*n*) = *n*/*L* − (*n* − 1)/*L* = 1/*L* and if *l* < *n*, Δ*H*(*n*) = *l*/*L* − *l*/*L* = 0.

#### 2.1.2. Estimate Benefit

There are two cases for acquiring of new cache blocks: (1) a demand access that misses in the cache; (2) prefetching. Because demand access is undeniable, our algorithm assigns its benefit as infinite value. In sequential access pattern and loop period of lopping access pattern, blocks are accessed contiguously. A prefetching can be performed to avoid future access to disks. If the prefetched data hits, prefetching can save the time of several disk I/O operations. If the prefetched data do not access, the cost of prefetching is the time to transfer the prefetched blocks. Thus, the benefit of prefetching can be describe as expression (3), where *P* is the probability of accessing prefetched blocks, *m* is the number of access of prefetched blocks, *D* is the degree of prefetching, and *B* is the bandwidth of disk:

(3) $$\begin{array}{c} \text{benefit}=P\cdot m\cdot T_{\text{disk}}-\left(1-P\right)\frac{D}{B}. \end{array}$$

#### 2.1.3. Block Replacement

We use the notion of marginal gains defined in previous works [9] to denote the change of performance with migration of a cache block which takes a cache block from supplier to customer. Marginal gain is defined as

(4) $$\begin{array}{c} \text{MG}\left(n\right)=\text{benefit}\left(P_{i}\right)-\text{cost}\left(P_{j}\right). \end{array}$$

The benefit to partition *P*_*i*_ (consumer) is computed with expression (3) and the cost to partition *P*_*j*_ (supplier) is computed according to its access patterns which we discussed above. When free blocks are available, the free cache blocks are allocated to partition as requested. When a demand access miss in the cache and the system has no free cache blocks left, our algorithm should take a cache block from appropriate partition. Our algorithm computes marginal gain for each partition and the partition with the largest marginal gain is chosen as the victim. To prevent the migration of cache blocks too frequently, a partition (*P*_*i*_) can consume a cache block from a partition (*P*_*j*_) only when it satisfies the constraint:

(5) $$\begin{array}{c} \text{MG}\left(n\right)\geq \delta , \end{array}$$

where *δ* is a threshold that should be chosen carefully. If there is no partition chosen as the victim partition, our algorithm will choose its own partition as supplier. Then, the replacement policy is invoked to evict a cache block from the supplier and allocate this cache block to the consumer.

### 2.2. Balance Cache Blocks across Partitions

Cache allocation based on benefit and cost analysis is a very effective approach to improve the utility of cache blocks. However it does not consider the effect of the partition size on the performance. Any supplier may become a new bottleneck partition as a result of shrinking too much cache blocks and incuring heavy performance degradation. For example, if a partition is set too small cache size then it may sharply increase in access delay as the change of workload characteristic, such as the workload change from sequence to random. In particular, it may lead to cache block thrashing. So, it is necessary to maintain a balance in distribution of cache block for stable performance. We propose a simple but effective approach to address this problem. If the workload on partition is growing heavy, it should guarantee enough cache space to prevent rapid increase on access delay. In contrast, if the workload on partition is growing light, it can supply more cache blocks to the partition with larger access delay. How the accuracy estimates the size of cache space required in the next period is the key problem in the balance scheme. Our algorithm sets a lower bound of cache size for each partition and denotes it as *B*, which is adjusted with the change of workload. Recall that the partition is split into 2 parts: provision area and adjustment area. The cache blocks within the size *B* belong to the provision area and the other cache blocks belong to the adjustment area. The cache blocks of the provision area are provisioned to meet the requirement of minimum cache size in the next period. So, the shrinking of cache block is bounded by the lower bound. However, setting lower bound may reduce flexibility of allocation among partitions. For an extreme example, if the sum of all partitions' lower bound is equal to the total cache size, then there will be no cache allocation at all. Thus, the lower bound should be subjected to the following constraint:

(6) $$\begin{array}{c} \overset{N}{\underset{i=1}{\sum }}B_{i}<\alpha \cdot C, \end{array}$$

where *N* is total number of partitions, *C* is total cache size, and *α* is a reasonable compromise value, 0 < *α* < 1.

Our algorithm periodically adjusts lower bound of each partition. The periodic reallocation scheme triggers adjustment after *T* requests where *T* is a parameter of the scheme. For each adjustment, our algorithm uses a two-step process to compute the required cache size for provision area: coarse adjustment and fine adjustment. The responsibility of coarse adjustment is to determine the direction of adjustment which refers to adding or shrinking of the cache blocks. The responsibikity of fine adjustment is to determine the degree of adding or shrinking for each partition. The computing of the degree for partitions is to adapt to the changes of the workload.

In the first phase, our algorithm should identify the partitions that should be increased of cache space and the partition that has surplus cache space. The surplus cache space refers to the cache blocks that if removed from the partition then it will not lead the access delay to outweigh the average value. The goal of our algorithm is to make all partitions achieve approximate access delay and eliminate the bottleneck partition. Thus, our algorithm classifies the partitions into suppliers and consumers according to the delay of those partitions. Partitions with above-average delay are considered as consumers and their lower bound will increase by a basic amount of cache blocks denoted as *I*. The partitions with below-average delay are considered as suppliers and their lower bound decreases by *I* cache blocks.

In the second phase, our algorithm predicts the change of workload and computes appropriate bound size for provision area. Our prediction is based on history of accesses. As locality, we assume that the workload remains unchanged within a short period. We use the difference in delay (Δ*d*) between the delay in current period (*d*_1_) and the delay in previous period (*d*_2_) to predict the variation of workload in the next period. If the difference is positive, it indicates that the I/O activities of the workload are becoming heavier. Then, our algorithm enlarges the basic amount of cache blocks for preventing further degradation of performance. In contrast, if the difference is negative, it indicates that the workload is becoming lighter and has surplus cache space to other partitions. For a partition with heavier workload, the partition should get more cache blocks for provision. Our algorithm enlarges adjustment amount by multiplying *I* with *f*_+_, where *f*_+_ is scale factor and “+” means the positive difference, *f*_+_ ∈ (1,2). The bigger the Δ*d* is, the larger the *f*_+_ will be. The larger *f*_+_ means that the provision area will increase more cache blocks and the same as the lower bound. In order to compute the *i*_*t*_ *h* partition's normalized scale factor *f*_+_^*i*^, we first select maximum difference in delay Δ*d*_max⁡_ and minimum difference in delay Δ*d*_min⁡_ from all partitions. Then we normalize the difference in delay of *i*_*t*_ *h* partition Δ*d*_*i*_ into *f*_+_ by the following expression:

(7) $$\begin{array}{c} f_{+}^{i}=1+\frac{\Delta d_{i}-\Delta d_{\min}}{\Delta d_{\max}-\Delta d_{\min}},\;f_{+}\in \left(1,2\right). \end{array}$$

For the partition with lighter workload, our algorithm will shrink its cache space and compress adjustment amount by multiplying *I* with *f*_−_, where *f*_−_ is scale factor and “−” means the negative difference, *f*_−_ ∈ (0,1). As the *f*_+_, the smaller Δ*d* is, the smaller *f*_−_ will be. The smaller *f*_−_ means that the partition can supply more surplus cache blocks to other partitions. Our algorithm normalizes the Δ*d*_*i*_ into *f*_−_^*i*^ by following expression:

(8) $$\begin{array}{c} f_{-}^{i}=\frac{\Delta d_{i}-\Delta d_{\min}}{\Delta d_{\max}-\Delta d_{\min}},\;f_{-}\in \left(0,1\right). \end{array}$$

## 3. Experiment

This section evaluates our cache algorithm. We use FileBench [10] to generate synthetic workloads, which is widely used as benchmark in storage system, such as [11, 12]. We built a simulator that implements Forney's algorithm, Liton's algorithm, and our algorithm. We also interfaced the Disksim 4.0 [13], an accurate disk simulator, to simulate the disk behaviors. The disk drive we modeled is the IBM 9LZX. As in [1, 2], we age its performance over a range of years to achieve heterogeneity, as shown in Table 2. The data layout policy uses RAID-0 (Redundant Array of Independent Disks) with 4 disks.

### 3.1. Performance Comparison

**Figure 3:**
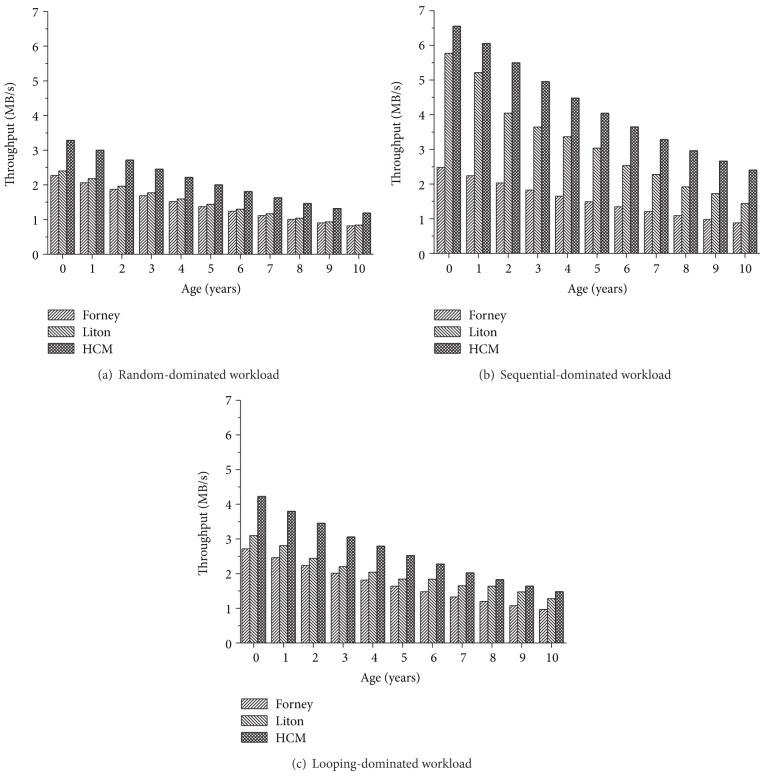
Throughput of the HCM and Forney algorithm with varying ages of the slow disk.

Figure 3 shows the throughput of Forney's, Liton's, and HCM algorithms when we vary the age of a disk ranging from 0 to 10 (step by one). Because the major effort of our algorithm to improve system performance is benefit-cost allocation based on reference type, we generate three types of workloads: random-dominated, sequential-dominated, and looping-dominated. The percentage of dominated type in workload is 60% and the other two types are 20%, respectively. We selected the cache size to be 100 MB for all experiments.

The results of Figure 3 show that the HCM algorithm outperforms Forney's algorithm and Liton's algorithm under all three workloads. For random-dominated workload, the HCM algorithm outperforms Forney's algorithm by 44.8% to 46.2% in throughput (on average 45.8%) and Liton's algorithm by 36.9% o 41.6% in throughput (on average 39.3%). For sequential-dominated workload, the HCM algorithm outperforms Forney's algorithm by 164.3% to 172.5% in throughput (on average 170.9%) and Liton's algorithm by 13.6% to 67.2% in throughput (on average 39.4%). For looping-dominated workload, the HCM algorithm outperforms Forney's algorithm by 52.1% to 54.7% in throughput (on average 53.4%) and Liton's algorithm by 11.3% to 41.5% in throughput (on average 28.4%). The major advantage of our algorithm is that the benefit-cost-aware replacement policy based on access pattern significantly improves the utility of the cache blocks. Compared with Forney's and Liton's algorithms, the HCM algorithm replaces the access delay with the difference between marginal benefit and cost as the allocation metric and ensures every allocation with the best utility. Furthermore, different with the Forney's and Liton's algorithms, the HCM algorithm will perform the prefetching when detecting sequential accesses. So, the HCM algorithm can obtain much more improvement under the sequential-dominated workload than under the random-dominated and looping-dominated workloads.

**Figure 4:**
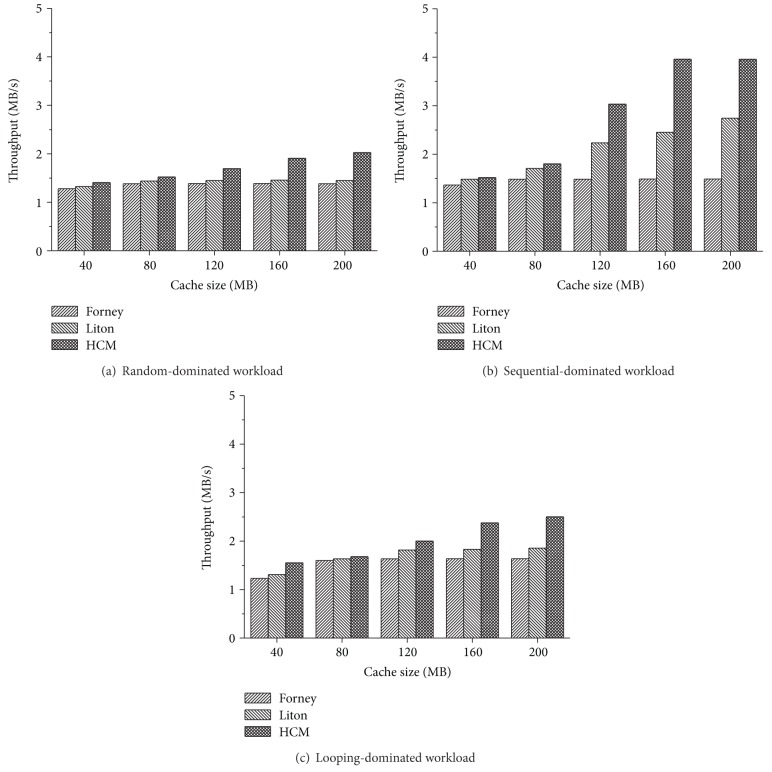
Throughput of the HCM and Forney algorithm with varying cache sizes.

To further demonstrate the effect of the HCM algorithm on utility, we design another set of experiments to compare the throughput of Forney's, Liton's and the HCM algorithms under different cache sizes. We vary the cache size from 40 MB to 200 MB (step by 40 MB). From the result of Figure 4, we can observe that the threshold for cache size exists in all three algorithms. In the HCM algorithm, the performance shows almost no improvement once cache size is beyond 160 MB. However, the threshold of the Forney algorithm is about 80 MB and the Liton algorithm is about 120 MB which is smaller than HCM algorithm. The second observation we make from Figure 4 is that the HCM algorithm obtains better results in throughput than the other two algorithms. For random-dominated workload, the HCM algorithm outperforms Forney's algorithm by 10.0% (40 MB) to 46.4% (200 MB) in throughput (on average 25.3%) and Liton's algorithm by 5.99% (80 MB) to 39.8% (200 MB) in throughput (on average 20.0%). For sequential-dominated workload, the HCM algorithm outperforms Forney's algorithm by 11.4% (40 MB) to 166.1% (200 MB) in throughput (on average 93.9%) and Liton's algorithm by 2.2% (40 MB) to 61.4% (160 MB) in throughput (on average 29.9%). For looping-dominated workload, the HCM algorithm outperforms Forney's algorithm by 5.0% to 52.9% in throughput (on average 30.4%) and Liton's algorithm by 2.7% (80 MB) to 34.8% (200 MB) in throughput (on average 19.2%). From these results, we can find that the larger the cache is, the more improvement the HCM algorithm will obtain.

### 3.2. Effect of Balance Scheme

To demonstrate the effect of balance scheme on performance improvement, we have compared the throughput under different imbalance workloads for three algorithms: Forney's algorithm, the HCM algorithm without balance scheme, and the HCM algorithm with balance scheme. The imbalance ratio of the workload is expressed as a percentage. For example, if the imbalance ratio of a workload is 20%, then the 20% of the workload will access to slow devices and the 80% of the workload will access to fast devices. In these experiments, the imbalance ratio of the workload is 5%, 15%, 20%, 25%, 30%, 60%, and 90%, respectively.

**Figure 5:**
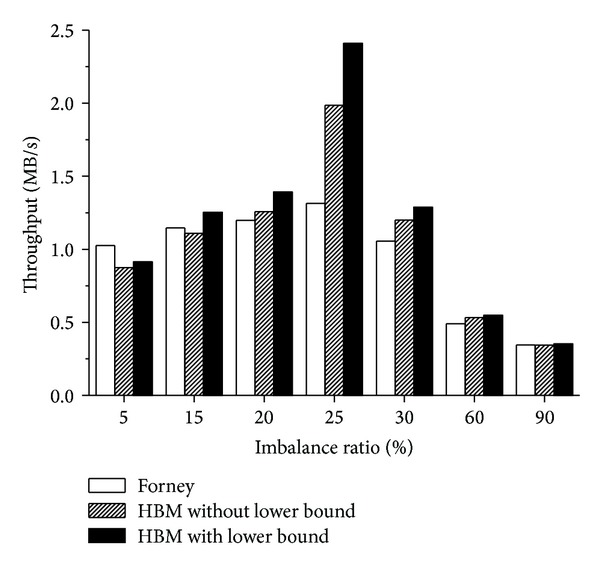
Throughput of Forney, HCM with and without balance scheme under imbalance workload.

We can find several observations from these results which were given in Figure 5. The first observation is that the HCM algorithm with the balance scheme obtains higher throughput than the other two algorithms for most workloads (only except the 5% case of Forney's algorithm). The HCM algorithm with balance scheme outperforms Forney's algorithm by −10.9% to 83.4% in throughput (on average 19.1%) and outperforms the HCM without balance scheme by 2.5% to 21.3% in throughput (on average 8.9%). We found that the HCM algorithm with or without balance scheme obtained worse improvement at the extreme imbalance ratio (e.g., 5% and 90%) than other imbalance ratios. That is because in such extreme distribution of accesses among partitions, the provision cache blocks in light-loaded partitions are accessed little and decrease the overall performance. The second observation is that the HCM algorithm with balance scheme obtains larger improvement with decrease of imbalance degree. In our experiments, the disk array was composed of 4 disks. So, the 25% point is more load balancing than other points. The HCM algorithm with balance scheme obtained 83.4% improvement in throughput than Forney's algorithm and 21.3% than the HCM without balance scheme at the 25% point. Both improvements are the largest among all imbalance points. The load balancing algorithms are widely adopted in the modern storage system to guarantee that the accesses are balanced distribution among all devices [14–16]. These experiments proved that the balance scheme can improve the performance and is suitable to the practical storage systems.

## 4. Related Work

There have been works on cost-aware caching in many areas, such as web caching and main memory. The web cache community has widely researched cost-aware caching [17–19]. In web caching, the data blocks differ from fixed-size blocks used in storage systems where all data blocks have a uniform size and uniform cost.

There are also many papers that focus on main memory adapting to the cost-aware caching. Kim et al. [6] had proposed a unified buffer management scheme (UBM) for main memory. The UBM first detects the access pattern and stores the detected blocks in separate partitions of cache. The allocation of cache blocks is tackled with the use of the marginal gains. Choi et al. [7] had proposed similar cache management scheme with Kim, which also exploits detecting of the access pattern and analysis of the marginal gain. Yadgar [8] had proposed Karma, which uses the marginal gain of cache blocks in multilevel cache. Karma leverages application hints to analyze the hit ratio (performance) and make informed allocation and make replacement decisions in all cache levels.

There are also many papers that focus on the effect of cache in disk array. Bairavasundaram et al. [20] had proposed a noninvasive exclusive caching mechanism for raids. X-ray achieves a high degree of exclusivity by gray-box methods. Baek and Park [21] had proposed a prefetching scheme with adaptive cache culling for striped disk arrays. The prefetching scheme provides low prefetching cost and evicts prefetched block at proper time by using feedback and maximizes the hit ratio of prefetched block and caching block. Wan et al. [22] had proposed an asymmetric cache to boost the performance of disk arrays under faulty conditions. The basic idea is to give higher priority to cache the blocks on the faulty disks and reduce the I/Os directed to the faulty disks.

## 5. Conclusion

In this paper, we have identified a series of problems in cache algorithms in heterogeneous storage systems and proposed a novel cache algorithm called HCM. It incorporates benefit-cost analysis in the cache allocation, which aims to maximize the utilization of caches. Furthermore, it achieves balanced system utilization through dynamic adjustment of lower bound of cache size for each partition. Simulation results show that our algorithm is effective and gives much higher throughput than Forney's algorithm.

## Figures and Tables

**Table 1:** Classification of cost-aware cache algorithms for heterogeneous storage systems.

	Balance cache block across partitions	Utility of cache block
Forney algorithm	√	
Utility-based algorithm		√
HCM algorithm	√	√

**Table 2:** We model different performance of disks based on IBM 9LZX manufactured in progressively order years.

Age (years)	Bandwidth (MB/s)	Seek avg. (ms)	Rotation avg. (ms)
0	20.0	5.30	3.00
1	14.3	5.89	3.33
2	10.2	6.54	3.69
3	7.29	7.27	4.11
4	5.21	8.08	4.56
5	3.72	8.98	5.07
6	2.66	9.97	5.63
7	1.90	11.1	6.26
8	1.36	12.3	6.96
9	0.97	13.7	7.73
10	0.69	15.2	8.59
